# The combined effects of coping and pain interference on army readiness

**DOI:** 10.3389/fpain.2023.1175574

**Published:** 2023-08-15

**Authors:** Jessica L. Kegel, Josh B. Kazman, Daniel R. Clifton, Peter Emanuele, D. Alan Nelson, Patricia A. Deuster

**Affiliations:** ^1^ Consortium for Health and Military Performance, Department of Military and Emergency Medicine, F. Edward Hébert School of Medicine, Uniformed Services University, Bethesda, MD, United States; ^2^ The Henry M. Jackson Foundation for the Advancement of Military Medicine, Inc., Bethesda, MD, United States

**Keywords:** pain, coping, military, psychosocial factors, resilience

## Abstract

**Introduction:**

Chronic pain and associated interference with daily activities are common in the military and impact Force readiness. Chronic pain affects one-third of service members and is a leading cause of medical non-readiness (MNR) in the military. Research suggests that underlying psychological mechanisms related to trait coping styles and pain interference (PI) affect functional outcomes, but little research exists examining this relationship within an Army population. The purpose of this study was to examine the combined effects of PI and coping on U.S. Army soldier readiness by using annual well-being data from the Global Assessment Tool (GAT) and medical non-readiness (MNR) based on duty restriction records.

**Methods:**

The sample comprised 866,379 soldiers who completed the GAT between 2014 and 2017 with no duty restrictions at the time of baseline GAT completion; subjects were observed through 2018 for duty restrictions. Parametric survival regression models with a Weibull distribution predicted demographic-adjusted hazards of MNR by dichotomized PI (no PI/PI) and beneficial/non-beneficial use of GAT coping components (good coping, bad coping, catastrophizing-flexibility, and catastrophizing-hopelessness). Incident MNR was evaluated for all duty restrictions, and stratified by selected body systems (upper extremity, lower extremity, psychiatric).

**Results:**

Among soldiers with PI, hazards were higher in those reporting non-beneficial coping styles (bad coping, hopelessness) and lower in those reporting beneficial coping styles (good coping, flexibility). Across all coping styles, PI/coping interactions were particularly strong for catastrophizing-hopelessness and when examining MNR from psychiatric conditions.

**Discussion:**

These findings suggest some synergistic associations between pain and coping that may impact pain-related occupational disability. Coping skills may be an effective interventional target for chronic pain reduction/prevention within military programs, such as the Master Resilience Training Course offered to soldiers in the Army. Further research should assess whether early coping style interventions can reduce pain-related outcomes.

## Introduction

Chronic pain is a leading health burden in the United States and results in over $500 billion in lost productivity and healthcare costs (1); it affects about 15% of the workforce (2). Chronic pain is particularly problematic for military service members, who have physically demanding tasks and need to maintain medical readiness. Over one-third of service members will experience chronic pain, based on diagnoses and patterns of healthcare utilization (3, 4). Chronic pain, typically related to musculoskeletal injuries, is also a leading cause of medical non-readiness (MNR), which leads to lost duty time and early retirement (5).

The purpose of this study was to examine the role of coping in long-term outcomes associated with pain interference in the general Army population. Population-based efforts to prevent pain and to mitigate its effects focus on hazard reduction, workplace policies, and healthcare access (6). At the population level, little is known about chronic pain prevention. However, paradigms for coping with psychological stress may provide some insight (7), particularly because the maintenance of chronic pain likely involves psychological mechanisms (8, 9).

Pain, by its nature, is an intense stimulus, which demands a reaction and easily produces a learned response (8, 10). When these responses fail to resolve the pain, they threaten to perpetuate the pain and its disabling effects. In addressing this negative feedback loop, psychological perspectives on chronic pain focus on fear-learning and avoidance, pain-catastrophizing (8, 11), rumination and anxiety (10), and negative cognitions (12) – characteristics that correlate with pain and pain-related outcomes, and are frequent targets in long-term pain treatment.

Responses to pain can also be conceptualized using a broader diathesis-stress model (13), which is often applied to life stressors and psychopathology (14, 15). In the diathesis-stress model, the pathological effect of a stressor is a function of its severity and the individual's capacity to cope with it, both of which dynamically change over time. A person's capacity to cope with a given stressor is dictated by various characteristics, such as genetics, psychosocial traits (e.g., resiliency (16) coping styles (17)), learned responses to specific stressors (18, 19), and contextual factors (20, 21). Psychosocial traits that are particularly relevant to the stress-response include resiliency [the ability to adapt to adversity (16)], coping styles [thoughts and behaviors used to manage stress (22)], and explanatory styles [habitual explanations for bad events (23)]. These traits directly correlate with positive outcomes [e.g., health, wellbeing (24, 25)] and negative outcomes [e.g., behavioral and psychiatric problems (24, 26)]; they are also particularly relevant for individuals who face chronic stress and adversity (27), such as persistent pain (13).

Per the diathesis-stress model, we tested the hypothesis that coping styles would moderate the association between pain and negative long-term outcomes. Support for this hypothesis would imply that interventions that focus on coping styles could mitigate pain-related morbidity. We used retrospective survey data, which included self-reported pain and select coping/explanatory styles. By using a large general Army population sample, we were able to link the data to MNR, a long-term functional outcome, which flags soldiers who are not medically ready to deploy or perform essential tasks. Importantly, this sample provided many subjects with and without pain, and with and without select coping styles, allowing us to isolate the individual and joint effects of pain and coping.

## Methods

The study protocol was approved by the Institutional Review Board at the Uniformed Services University in Bethesda, MD. De-identified data related to Army personnel were accessed through the Person-Event Data Environment, a data repository maintained by the Army Analytics Group Research Facilitation Laboratory. Only respondents that consented to have their responses used for research purposes were included in the analytic sample.

Data were also collected from Global Assessment Tool (GAT) responses. The GAT is an annual self-report survey that collects “fitness” data from various domains including physical, psychological, family, social, and spiritual health. Upon completion of the required survey, soldiers can consent to allow their data be used for research purposes. GAT data are available for about 40% of all soldiers, as previously described (28).

GAT data were combined with administrative data from the Defense Manpower Data Center (DMDC) and Medical Operational Data Systems, which contain medically-directed duty restrictions (or “profiles”). For the purpose of this study, we assessed data from 866,379 soldiers who completed a baseline GAT between 2014 and 2017, consented to their records being used for research, and had no duty restrictions at the time of the baseline GAT. Soldiers were then screened for incident MNR through 2018. Median observation time per soldier was 33 months.

### Demographics

Age, sex, marital status, career path (enlisted vs. officer), and service duration were captured from personnel records made available through the DMDC.

### Duty restrictions

MNR, our dependent variable, was based on permanent duty restrictions from regular medical evaluations and captured in eProfile records. Most duty restrictions are categorized by body system (commonly referred to as “PULHES”) (29). The PULHES body systems include physical capacity/stamina (P), upper extremities (U), lower extremities (L), hearing (H), eyes (E), and psychiatric (S). Our main outcome was any permanent MNR. Additional analyses examined MNR with limitations in upper extremities, lower extremities, and psychiatric diagnoses.

### Coping styles

The GAT was used to assess coping styles. GAT scales are a combination of previously published scales and items adapted or developed specifically for the GAT (24, 30). The four GAT scales used in this study were good coping (6 items), bad coping (3 items), catastrophizing-flexibility (3 items), and catastrophizing-helplessness (3 items). All scales demonstrated adequate levels of reliability in our sample and in similar samples, with omega values ranging from 0.71 (catastrophizing-flexibility) to 0.84 (good coping) (24, 31). We collectively refer to those scales as “coping styles”.

Catastrophizing, which is a tendency to assume the worst, is measured using two sub-scales – flexibility (e.g., “I am good at changing myself to adjust to changes in my life”) and hopelessness (e.g., “When bad things happen to me, I expect more bad things to happen.”). These items are based on the work of Peterson et al. on explanatory styles and trauma (19), which emerged out of earlier learned helplessness models (14, 18).

The good and bad coping scales are based on Carver et al.'s brief coping style scales (32). The original versions of the scales asked about methods of responding to a specific stressor, which might have been specific to a study (e.g., natural disaster) or a person (e.g., “think about a stressful event in the prior month”). Sub-scales captured 15 distinct coping techniques (e.g., positive re-interpretation, mental disengagement).

On the GAT, the coping scales ask about responses to stress in general, and then group the coping techniques into “good” or “bad” coping style scales. Items on the good coping scale ask about acceptance, problem solving, and emotional control (e.g., “For things I cannot change, I accept them and move on”). Items on the bad coping scale ask about avoidance, isolation, and bottling up emotions (e.g., “When something stresses me out, I try to avoid it or not think about it”). Because of these adaptations, the GAT's coping scales could be considered general dispositional coping styles (17, 32), rather than specific coping techniques.

For all four GAT scales, items were answered on Likert-type scales, with item response codes of one (“Not like me at all”), two (“A little like me”), three (“Somewhat like me”), four (“Mostly like me”), and five (“Very much like me”). The scores were averaged, and then dichotomized based on low usage (scores of one or two) or moderate to high usage (scores of three to five). To avoid confusing statements below (e.g., “low good coping”/“high bad coping”), we adopted the term “beneficial” to indicate a “good” or positive score on the scale (i.e., high good coping; high flexibility; low bad coping; low hopelessness) and “non-beneficial” to indicate a “bad” or negative score on the scale. In all analyses, “beneficial” scores were coded as 0, or the reference condition, and “non-beneficial” scores were coded as 1, or the indicator condition. This scheme standardized our interaction analyses (33), although it does differ from most prior work with the GAT where all scales are coded so that “higher” scores are “better” (24).

### Pain interference (PI)

Soldiers were asked to indicate the level of PI in usual activities during the past 30 days on a scale of zero (“no pain”) to ten (“as bad as it could be, nothing else matters”). For this analysis, scores were dichotomized to indicate no to some pain that does not interfere with usual activities (scores of zero to four) or some to extreme pain that does interfere with usual activities (scores of five–ten).

### Analyses

Analyses were completed using Stata/MP 15.1 (StataCorp LLC. Released 2017. Station, TX). Parametric survival regression models with a Weibull distribution were used to predict incident MNR. Different parallel models were executed for each coping scale. Unadjusted hazard ratios (HR) and HR adjusted for gender, age, service duration, career path (enlisted vs. officer), marital status, and GAT social health domain are reported. In each model, individuals were classified into one of four groups: a non-exposure group (no PI/beneficial coping), which served as the reference group; a doubly exposed group (PI/non-beneficial coping); and two single-exposure groups (PI/beneficial coping; no PI/non-beneficial coping). Next, we examined interaction analyses to determine whether risk for individuals with both factors, or the doubly exposed group (i.e., PI/non-beneficial coping), was greater than would be expected from the combination of the two single-exposure groups.

The nature of the interaction between PI and non-beneficial coping was determined by examining: (1) adjusted hazard ratios across the three exposure groups, and (2) Relative Excess Risk due to Interaction (RERI) and Synergy (S) indexes (34). The RERI and S are complementary measures for interpreting whether the joint effect of two exposures is greater than the expected effect from the combination of individual exposures. In other words, is the effect of having both PI and non-beneficial coping greater than the sum of their parts? RERI is computed by taking the risk for the doubly exposed group (RR_A + B+_), subtracting risk for the two single-exposure groups (RR_A − B+_, RR_A + B−_), and then adding 1; values over 0 indicate positive interaction. *S* is computed from the same risk estimates, but as a ratio:

$$S\;=\;\;\frac{RR_{A+B+}-1}{\left(R_{A+B-}-1)\;+\;(R_{A-B+}-1\right)}$$

*S* values above 1 indicate positive interaction.

Confidence intervals for RERI and *S* were estimated using STATA's post-estimates non-linear combinations command.

## Results

### Demographics

The sample of 866,379 soldiers had an average age of 29 years and an average service duration of 9 years (Table 1). Most of the sample was enlisted (79.6%), male (82.7%), and not married (61.8%). During the MNR observation time, approximately 5% of soldiers received a permanent duty profile resulting in duty restrictions.

**Table 1:** Demographics (*n* = 866,379).

Demographics	Frequency (%)/Mean (SD)
Age (years)	28.6 (9.1)
Gender	
Male	82.7%
Female	17.3%
Marital Status	
Not Married	61.8%
Married	38.2%
Career Path	
Enlisted	79.6%
Officer	20.4%
Mean service duration, years (SD)	9.3 (9.4)
Permanent Duty Profile	
No	95.3%
Yes	4.7%
Good Coping/PI Groups	
Beneficial coping/No PI	44.0%
Beneficial coping/PI	6.9%
Non-beneficial coping/No PI	37.9%
Non-beneficial coping/PI	11.2%
Bad Coping/PI Groups	
Beneficial coping/No PI	58.9%
Beneficial coping/PI	12.4%
Non-beneficial coping/No PI	23.0%
Non-beneficial coping/PI	5.7%
Catastrophizing-Flexibility/PI Groups	
Beneficial coping/No PI	56.4%
Beneficial coping/PI	10.0%
Non-beneficial coping/No PI	25.4%
Non-beneficial coping/PI	8.1%
Catastrophizing-Helplessness/PI Groups	
Beneficial coping/No PI	79.6%
Beneficial coping/PI	17.1%
Non-beneficial coping/No PI	2.3%
Non-beneficial coping/PI	1.0%

### Good coping groups

Hazard ratios (HR) derived from the survival analyses indicate MNR risk was lower among soldiers who reported beneficial coping/PI (unadjusted HR = 3.9, adjusted HR = 2.9, *p* < 0.05; Table 2) than those who reported non-beneficial coping/PI (unadjusted HR = 5.1, adjusted HR = 3.0, *p* < 0.05) compared to the beneficial coping/No PI group. Soldiers who reported non-beneficial/no PI had a significantly higher risk of duty restrictions compared to the beneficial coping/No PI group in unadjusted models (unadjusted HR = 1.2, *p* < 0.05), but these results were no longer significant when adjusted for covariates.

**Table 2:** Associations between coping, pain interference, and medical non-readiness risk.

Coping/Pain groups	Hazard ratios (95% CI)		Interaction metrics (95% CI)	
	Unadjusted	Adjusted	RERI	*S*
High Good Coping/No PI			0.1 (0, 0.2)	1.1 (1.0, 1.1)*
(*Reference*)				
Beneficial/PI	3.9 (3.8, 4.1)*	2.9 (2.8, 3.0)*		
Non-beneficial/No PI	1.2 (1.2, 1.3)*	1.0 (1.0, 1.0)		
Non-beneficial/PI	5.1 (5.0, 5.2)*	3.0 (2.9, 3.1)*		
Low Bad Coping/No PI			0.4 (0.3, 0.5)*	1.2 (1.1, 1.3)*
(*Reference*)				
Beneficial/PI	4.1 (4.0, 4.2)*	2.9 (2.8, 3)*		
Non-beneficial/No PI	1.0 (1.0, 1.1)*	1.1 (1.0, 1.1)*		
Non-beneficial/PI	4.7 (4.5, 4.8)*	3.3 (3.2, 3.4)*		
High Flexibility/No PI			0.3 (0.1, 0.4)*	1.1 (1.1, 1.2)*
(*Reference*)				
Beneficial/PI	4.0 (3.9, 4.1)*	2.3 (2.9, 3)*		
Non-beneficial/No PI	1.3 (1.3, 1.4)*	1.2 (1.1, 1.2)*		
Non-beneficial/PI	5.4 (5.3, 5.6)*	3.4 (3.2, 3.5)*		
Low Hopelessness/No PI			0.6 (0.3, 1.0)*	1.3 (1.1, 1.4)*
(*Reference*)				
Beneficial/PI	4.1 (4.1, 4.2)*	2.9 (2.9, 3.0)*		
Non-beneficial/No PI	1.6 (1.5, 1.7)	1.5 (1.4, 1.7)*		
Non-beneficial/PI	6.6 (6.2, 7)*	4.1 (3.8, 4.4)*		

PI, pain interference; RERI, relative excess risk due to interaction; S, synergy index. Survival analysis models have been adjusted for the following covariates: gender, age, service duration, career path (enlisted vs. officer), marital status, and the GAT social health domain. Separate models were executed for each coping style.

* Results are significant at *p* < 0.05.

### Bad coping groups

For bad coping, MNR risk was lower among soldiers who reported beneficial coping/PI (unadjusted HR = 4.1, adjusted HR = 2.9, *p* < 0.05; Table 2) than those who reported non-beneficial/PI (unadjusted HR = 4.7, adjusted HR = 3.3, *p* < 0.05) compared to the beneficial coping/no PI group. Soldiers who reported non-beneficial coping/no PI had a statistically significantly higher risk of MNR compared to the beneficial coping/no PI group, but the magnitude of the risk was minimal (unadjusted HR = 1.0, adjusted HR = 1.1, *p* < 0.05).

### Catastrophizing-Flexibility groups

For catastrophizing-flexibility, MNR risk was lower among soldiers who reported beneficial coping/PI (unadjusted HR = 4.0, adjusted HR = 2.3, *p* < 0.05; Table 2) than those who reported non-beneficial coping/PI (unadjusted HR = 5.4, adjusted HR = 3.4, *p* < 0.05) compared to the beneficial coping/No PI group. Soldiers who reported non-beneficial coping/no PI had a greater risk of MNR compared to the beneficial coping/No PI group (unadjusted HR = 1.3, adjusted HR = 1.2, *p* < 0.05).

### Catastrophizing-Helplessness groups

For catastrophizing-helplessness, MNR risk was lower among soldiers who reported beneficial coping/PI (unadjusted HR = 4.1, adjusted HR = 2.9, *p* < 0.05; Table 2) than those who reported non-beneficial coping/PI (unadjusted HR = 6.6, adjusted HR = 4.1, *p* < 0.05) compared to the beneficial coping/no PI group. Soldiers who reported non-beneficial coping/no PI had a greater risk of MNR compared to the beneficial coping/no PI group (unadjusted HR = 1.6, adjusted HR = 1.5, *p* < 0.05).

All coping by PI Interaction metrics were in the expected directions (RERI > 1; *S* > 1; Table 2), although they were stronger for the catastrophizing-helplessness model than for the other coping styles.

### Specific body systems

Survival analyses indicated an increase in magnitude of HR when MNR was specifically related to upper extremities, lower extremities (SupplementaryA), and psychiatric diagnoses (Table 3). Risks related to psychiatric diagnoses were most notable and had the stronger coping/PI interaction metrics (i.e., RERI and *S*) than other types of MNR. The risk of psychiatric-related MNR was lowest among soldiers who reported high catastrophizing-flexibility (unadjusted HR = 3.3, adjusted HR = 2.5, *p* < 0.05; Table 3) and low catastrophizing-hopelessness (unadjusted HR = 3.7, adjusted HR = 2.5, *p* < 0.05) than those who reported low catastrophizing-flexibility (unadjusted HR = 7.8, adjusted HR = 3.8, *p* < 0.05) and high catastrophizing-helplessness (unadjusted HR = 11.3, adjusted HR = 4.8, *p* < 0.05) compared to the beneficial coping/no PI groups. Soldiers who reported low catastrophizing-flexibility (unadjusted HR = 2.2, adjusted HR = 1.5, *p* < 0.05) and high catastrophizing-helplessness (unadjusted HR = 3.1, adjusted HR = 2.3, *p* < 0.05) had a significantly higher risk of psychiatric-related MNR compared to the beneficial coping/no PI groups.

**Table 3:** Associations between coping, pain interference, and medical non-readiness risk due to psychiatric conditions.

Coping/PI groups	Hazard ratios (95% CI)		Interaction metrics (95% CI)	
	Unadjusted	Adjusted	RERI	*S*
High Good Coping/No PI			0.6 (0.3, 0.8)*	1.4 (1.2, 1.6)*
(*Reference*)				
Beneficial/PI	3.0 (2.8, 3.3)*	2.3 (2.1, 2.5)*		
Non-beneficial/No PI	1.8 (1.7, 1.9)*	1.1 (1.1, 1.2)*		
Non-beneficial/PI	6.7 (6.4, 7.1)*	3.0 (2.7, 3.2)*		
Low Bad Coping/No PI			0.5 (0.3, 0.7)*	1.3 (1.1, 1.5)*
(*Reference*)				
Beneficial/PI	3.7 (3.5, 3.9)*	2.4 (2.3, 2.6)*		
Non-beneficial/No PI	1.3 (1.2, 1.4)*	1.2 (1.1, 1.3)*		
Non-beneficial/PI	5.3 (5.0, 5.6)*	3.1 (2.9, 3.4)*		
High Flexibility/No PI			0.8 (0.5, 1.0)*	1.4 (1.2, 1.6)*
(*Reference*)				
Beneficial/PI	3.3 (3.1, 3.6)*	2.5 (2.3, 2.7)*		
Non-beneficial/No PI	2.2 (2.0, 2.3)*	1.5 (1.4, 1.6)*		
Non-beneficial/PI	7.8 (7.3, 8.2)*	3.8 (3.5, 4.1)*		
Low Hopelessness/No PI			0.9 (0.3, 1.5)*	1.3 (1.1, 1.5)*
(*Reference*)				
Beneficial/PI	3.7 (3.5, 3.9)*	2.5 (2.4, 2.7)*		
Non-beneficial/No PI	3.1 (2.7, 3.4)	2.3 (2.1, 2.7)*		
Non-beneficial/PI	11.3 (10.3, 12.4)*	4.8 (4.2, 5.4)*		

PI, pain interference; RERI, relative excess risk due to interaction; S, synergy index. Survival analysis models have been adjusted for the following covariates: gender, age, service duration, career path (enlisted vs. officer), marital status, and the GAT social health domain. Separate models were executed for each coping style.

* Results are significant at *p* < 0.05.

## Discussion

The results of our study support the application of the diathesis-stress model, using coping styles (14, 15), to pain (13), as we found that the ability for PI to predict MNR depended on an individual's coping style. In our results, PI and non-beneficial coping styles each individually entailed some level of MNR risk; as expected, soldiers who reported both PI and non-beneficial coping had the greatest MNR risk compared to soldiers reporting neither or one of these factors. However, for most models, we identified a synergistic effect bewteen PI and non-beneficial coping, indicating that their joint effect was greater than what would be expected from the sum of their individual effects combined. This synergistic effect was magnified in models that used psychiatric-based MNR as an outcome and in models that examined hopelessness as a coping style. These findings have important implications for primary and secondary prevention of pain outcomes. First, however, it is necessary to place them in the context of literature on pain, coping, and the diathesis stress model.

### Pain, coping, and the diathesis-stress model

To contextualize our findings, it is necessary to elaborate on the GAT and to distinguish between a few lines of research with nomological overlap – coping in general, explanatory style, and coping with pain.

The GAT survey is a component of the Comprehsenive Solider and Family Fitness (CSF) program, which originated in response to behavioral health problems after the wars in Iraq and Afghanistan (35). The constructs included in the GAT were selected and honed to provide soldiers personal feedback about their fitness and psychosocial functioning; a secondary intention was to assess the wellbeing of the Army and facilitate epidemiological research (36). Its scales were mostly based on previously published items, although some scales and items customized to suit the GAT's purpose.

Four scales on the GAT examine constructs that are explicitly defined by how people respond to stress. In the previous source material for the GAT, these scales were called “good coping”, “bad coping”, “catastrophizing-helplessness”, and “catastrophizing-flexibility”. The two coping scales partially reflect more conventional measures of coping “styles”, which are strategies people use to respond to stressors (17). They derive from research demonstrating that the acute effects of a given stressor – such as an experimentally-induced stimuli, a large-scale tragedy, a life-event, or a daily hassle – are determined by how people cope with it (20, 32). Individuals tend to respond to different stressors with global attributes (e.g., using similar strategies across many stressors) and local attributes (e.g., unique to specific stressors) (17). As such, people likely employ individual strategies that vary by context and stressor, but also have more global coping “styles”, which resemble personality traits. Typically, individual coping strategies are not considered “good” or “bad” because they frequently depend on the context (e.g., humor might be appropriate for some stressors, but inappropriate for others); however, more global coping styles, such as a tendency to bottle up emotions, can be a clear risk for more severe psychological symptoms.

The two catastrophizing scales measure explanatory styles, which are how people tend to explain or infer the reasons for why negative events occur to them (19). They are derived from a distinct line of research linking attributional styles – such as a tendency to assume negative events will keep on happening to you – to mood and anxiety disorders (22).

These two types of constructs – coping and explanatory style – partially resemble constructs that are central in pain treatment and prognosis, but they differ in key manners. Patients with pain frequently develop affective and cognitive techniques for coping with the pain; if the pain persists and these techniques become habituliazed, then they become key correlates and mediators of pain severity and morbidity (37–41). Such techniques may be adaptive (e.g., lifestyle changes, humor) or maladaptive (e.g., avoidance, rumination, catastrophizing). These techniques are also prognostic indicators, particularly for individuals who develop chronic pain. Treatments that target these techniques have consistently demonstrated success in moderatly reducing pain-related morbidity (42–44). Within clinical practice guidelines for chronic or musculoskeletal pain, psychosocial components often include targeting pain-coping or similar responses (e.g., catastrophizing), alongside screening for mental disorders (45, 46).

Not much is known about how general coping styles relate to specific approaches to coping with pain. It is certainly possible that when answering questions about their general coping and explanatory styles in response to stress, some soldiers with pain were thinking about pain-related stress. More generally, however, severe pain, as one of many stressors, may be detrimental to one's capacity to cope with a variety of life stressors. This relates to a central thesis of the diathesis stress model as applied to coping approaches – namely that, when faced with severe adversity, one has to consider the sum total of many stressors, and the personal capacity to deal with them globally (13, 14). A corollary is that coping styles for handling stress only become important once people face stress.

In line with this conceptualization, it is noteworthy that, conditioned on no PI, we found that most non-beneficial coping styles (i.e., low levels of “good coping” on the GAT and high levels of “bad coping”, and catastrophizing-inflexibility) posed minimal MNR risk, with adjusted hazard ratios around 1.0–1.2. It was only in the presence of PI that these attributes appreciably increased risk. These findings reflect the severity of PI-related stress, or, at the least, to PI's potential to serve as a single-item indicator for stress.

### Prevention and treatment

One implication of our findings is that general population techniques for enhancing resiliency or bolstering the stress response may be particularly relevant for patients with pain. In the Army, a longstanding program to prevent psychological problems and optimize performance uses a train-the-trainer model, whereby soldiers are incentivized to complete a 10-day Master Resiliency Training course to bolster the resiliency of themselves and their assigned units (47). The course skills intentionally overlap with most of the items measured in the GAT (30). The course is based on concurrent resiliency programs at the University of Pennsylvania, which approach resiliency as a modifiable skill and focus on practical habits to improve resiliency (47, 48). Research is limited on the impact of this course, however, early reports do support its efficacy for improving the resilience of attendees (49) and their assigned units (50).

Continued resiliency training and similar programs might reduce pain-related burden, particularly for long-term psychological problems. These programs might be particularly relevant for patients with chronic pain, but may also be justifiable among populations at high risk of PI, chronic pain, and psychopathology. The strong associations we observed for psychological outcomes also illustrates how these findings may be more important for subjects with pain who are at heightened risk for psychological disorders, in contrast to patients who are at risk for other chronic disorders. Within the military, mental disorders have become a leading disease burden, accounting for over half of all military hospital bed days (45, 51). As shown in our results, and consistent with the diathesis-stress model (15), emergent symptoms of PI secondary to a mental disorder may pose significant risk.

### Limitations

Our study was largely informed by models for chronic pain (7–9, 52), although it should be noted that not all individuals with PI will go on to develop chronic pain. In particular, chronic pain requires a duration of three months (53), whereas our PI variable was based on a duration of one month. We took this approach under the assumption that the long-term disabling effects of PI likely function in a similar manner as the disabling effects of chronic pain.

Our sample was also limited to soldiers who had both completed the GAT and consented for their records to be used. This process availed a large sample that is likely comparable to the general Army population (28), but it may have introduced some bias (e.g., if MNR risk was associated with the choice to consent).

Additionally, the subjective nature of the GAT introduces the possibility of self-report bias and potentially skewed results (54). Given that the primary predicting factors were perceived pain interference (an inherently subjective metric), and coping styles, which are traditionally measured subjectively, we believe that the self-report data collection method utilized on the GAT did not compromise the integrity of the study and is comparable to similar research on coping skills.

Lastly, our study was limited to a general population sample, in contrast to most studies examining psychological factors and pain-related burden, which used clinical research samples (e.g., patients with select diagnoses or from clinics) (8, 12). This approach was important, because it allowed us to examine health indicators and traits associated with select psychological factors, independent of PI. Moreover, the chronic effects of pain are likely similar across many conditions (7, 55).

## Conclusion

In a large general Army sample, both PI and non-beneficial coping styles increased the risk for MNR. The PI and coping styles are likely to have synergistic effects on MNR risk for soldiers who engage in catastrophizing and who have psychiatric-related conditions. Given the high prevalence of PI in our sample, and the heavy burden of chronic pain (3–5) and mental health conditions (56, 57) in military populations, additional studies and interventions are warranted for minimizing pain's long-term effects and improving associated outcomes.

## Data Availability

The data analyzed in this study is subject to the following licenses/restrictions: Data for this study were obtained via the Person-Data Environment (PDE), which requires DoD credentials, research approvals, and data agreements to access DoD-wide data. Therefore, data for this project can only be made available to select DoD groups with appropriate approvals. Related inquiries can be directed to the corresponding author. Requests to access these datasets should be directed to usarmy.pentagon.hqda-dusa.mbx.rfl-data-acquisition@army.mil.

## Disclaimer

The opinions and assertions expressed herein are those of the authors and do not reflect the official policy or position of the Uniformed Services University or the Department of Defense.
